# Influence of skin melanisation and ultraviolet radiation on biomarkers of systemic oxidative stress

**DOI:** 10.1016/j.freeradbiomed.2020.07.034

**Published:** 2020-11-20

**Authors:** Barbara B. Shih, Mark D. Farrar, Andy Vail, Donald Allan, Mu-Rong Chao, Chiung-Wen Hu, George D.D. Jones, Marcus S. Cooke, Lesley E. Rhodes

**Affiliations:** a Centre for Dermatology Research, Division of Musculoskeletal and Dermatological Sciences, School of Biological Sciences, Faculty of Biology Medicine and Health, The University of Manchester and Salford Royal NHS Foundation Trust, Manchester Academic Health Science Centre, Manchester, UK; b Centre for Biostatistics, Division of Population Health, Health Services Research & Primary Care, School of Health Sciences, Faculty of Biology Medicine and Health, The University of Manchester, Manchester Academic Health Science Centre, Manchester, UK; c Medical Physics Department, Salford Royal NHS Foundation Trust and The University of Manchester, Manchester Academic Health Science Centre, Manchester, UK; d Department of Occupational Safety and Health, Chung Shan Medical University, Taichung, 402, Taiwan; e Department of Public Health, Chung Shan Medical University, Taichung, 402, Taiwan; f Leicester Cancer Research Centre, University of Leicester, Leicester, UK; g Oxidative Stress Group, Department of Cell Biology, Microbiology and Molecular Biology University of South Florida, Tampa, FL, 33620, USA

**Keywords:** Oxidative stress, Melanin, Skin, Ultraviolet radiation, Nucleic acids, Biomarkers

## Abstract

Skin melanisation ranges widely across human populations. Melanin has antioxidant properties and also acts as a filter to solar ultraviolet radiation (UVR) incident upon the skin. In this study we firstly examined whether melanin level might influence baseline levels of systemic oxidative stress, in 65 humans in vivo from the same geographical area ranging from the lightest to darkest skin type (phototype I-VI). This was examined in winter-time (latitude 53.5°N). Remarkably, we found that urinary biomarkers of oxidatively-generated DNA damage (8-oxodG) and RNA damage (8-oxoGuo) were significantly correlated with skin lightness (L*), such that 14–15% of the variation in their baseline levels could be explained by skin colour. Next we exposed 15 humans at the extremes of skin melanisation to a simulated summer-time exposure of solar UVR (95% UVA, 5% UVB; dose standardised to sunburn threshold), following which they provided a sample of every urine void over the next five days. We found that UVR induced a small but significant increase in urinary 8-oxodG and 8-oxoGuo, with differing kinetics between skin types. Thus greater melanisation is associated with protection against systemic oxidative stress, which may reflect melanin's antioxidant properties, and solar UVR exposure also influences systemic oxidative stress levels in humans. These novel findings may have profound implications for human physiology and health.

## Highlights

• Biomarkers of systemic oxidative stress correlate with human skin lightness level.
• Skin colour contributes 14–15% variation in baseline urinary 8-oxodG and 8-oxoGuo.
• UVR exposure of the skin elevates biomarkers of systemic oxidative stress.
• Both oxidatively-damaged DNA (8-oxodG) and RNA (8-oxoGuo) increase post-UVR.
• Kinetics of urinary biomarker responses following UVR differ between skin types.

## Graphical abstract

**Figure:**
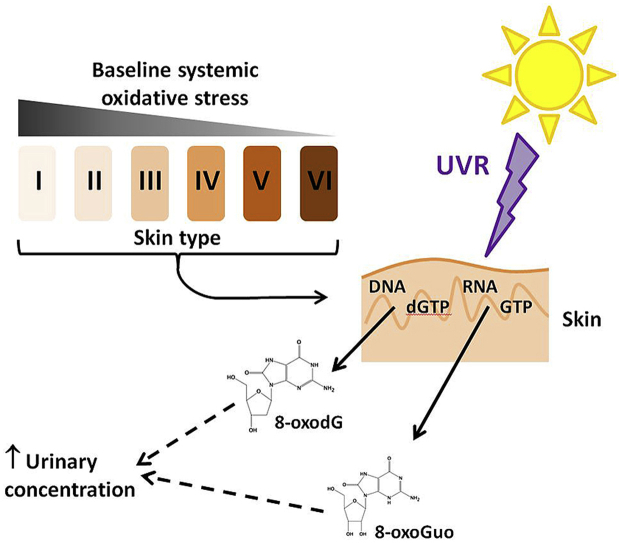

## 1 Introduction

Cutaneous melanin absorbs solar ultraviolet radiation (UVR), providing protection from skin cancer [1], although may also protect via its antioxidant properties. Reactive oxygen species (ROS) [2], and DNA strand breaks [3] both inversely correlate with melanocyte melanin levels *in vitro*. Levels of skin melanisation across the human skin colour range (phototype I-VI) also show differential distribution of the predominantly directly UVR-induced skin DNA lesion, the cyclobutane pyrimidine dimer (CPD), in vivo [4]. However, potential differences in the formation/repair of indirectly-generated, oxidatively-damaged DNA across human phototypes remains unexplored, despite their likely contribution to skin cancer development [5]. Moreover, oxidatively-generated DNA and RNA damage have wider significance as biomarkers of systemic oxidative stress, with potentially detrimental cellular effects [6]. They can be measured non-invasively via their urinary excretion [7], permitting multiple measurements and human biology investigation in vivo.

Oxidatively-generated DNA damage can be induced in skin cells through ROS derived from UVR-mediated photosensitization [8,9], the main oxidation product being 8-oxo-7,8-dihydro-2′-deoxyguanosine (8-oxodG) [[10], [11], [12]]. As 8-oxodG may mispair and misincorporate with adenine, failure to remove this DNA lesion can result in G→T and A→C transversion mutations [13], with implications for carcinogenesis. Indeed, oxidatively-generated DNA damage contributes ~8% of longer wavelength UVA-induced mutations [14]. However, ROS generation is not unique to UVR; they are produced by oxidative phosphorylation in mitochondria, and other endogenous and exogenous processes. Urinary 8-oxodG is proposed as a ‘whole-body’ biomarker of oxidative stress; while its precise origins are unclear, sanitisation of 8-oxodGTP from the dGTP nucleotide precursor pool appears the prime candidate [15].

UVR-induced RNA oxidation has been demonstrated in human skin fibroblasts *in vitro* [16]. Formation, repair, measurement, and biological consequences of oxidatively-generated RNA damage are less studied than for DNA, but information is emerging [17]. Similar to 8-oxodG, RNA oxidation products such as 8-oxo-7,8-dihydroguanosine (8-oxoGuo) are conveniently and sensitively measured in urine, with analogous origins i.e. the ribonucleotide precursor pool [18]. RNA oxidation is also suggested as a disease marker, offering different prognostic value from DNA oxidation markers [19,20]. In several experimental systems, levels of oxidatively-generated damage were higher in RNA than DNA [21]. However, we are unaware of studies examining urinary RNA in relation to melanin level or as a marker of UVR-induced oxidative stress.

Our objectives were: (1) evaluate baseline urinary 8-oxodG and 8-oxoGuo levels across the human phototypes, from I (light white skin) to VI (black skin), examining relationship to skin melanisation; (2) examine these biomarkers in the lightest and darkest phototypes after a single, sub-sunburn exposure to UVR simulating summer sunlight.

## 2 Material and methods

### 2.1 Study design

**Fig. 1:**
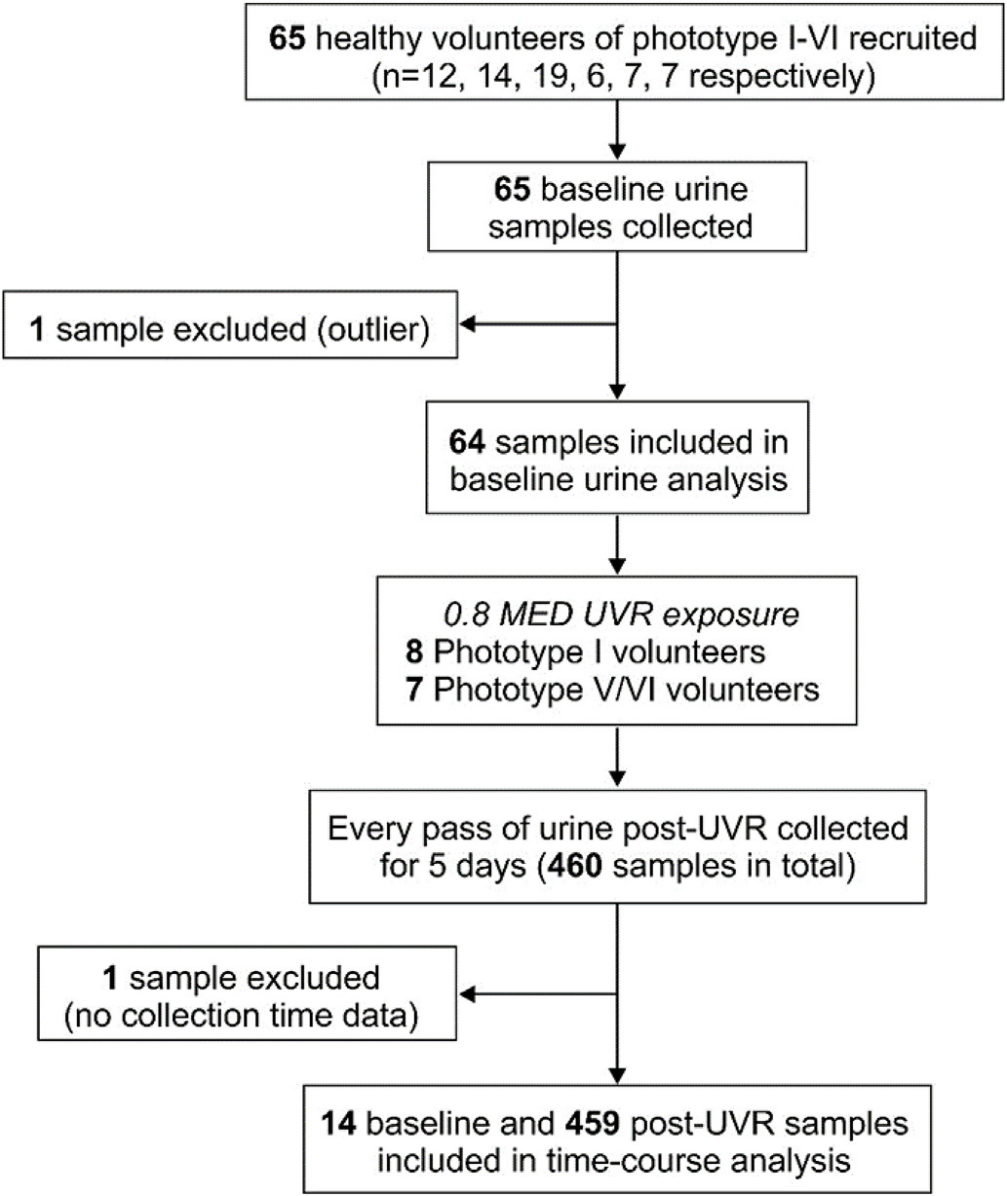
Flowchart of study protocol. Following detailed assessment of skin type, skin lightness and UVR-erythemal sensitivity, 65 volunteers of phototype I-VI (n = 12, 14, 19, 6, 7, 7, respectively) each provided a baseline urine sample. Volunteers with the lightest (phototype I, n = 8) and darkest (phototype V/VI, n = 7) skin were exposed to a UVR dose personalised to their sunburn threshold i.e. 80% of their minimal erythema dose (MED), and the temporal change in 8-oxodG and 8-oxoGuo evaluated through collection of every pass of urine over a five day period post-exposure. One participant had a baseline 8-oxodG 22 SD higher than the mean of the cohort and 55 times higher than the mean of the post-UVR samples from the same individual; this outlier was removed from the analysis.

The human study (Fig. 1) occurred at the Photobiology Unit, Dermatology Centre, Salford Royal Hospital, Manchester, UK (53.5°N), in November–March (2012–2013 or 2013–2014) when ambient UVR influence is minimal. Healthy volunteers, phototype I-VI, 20–49 y, from the Greater Manchester area, participated. Exclusions: history of skin cancer/photosensitivity, sunbathing/sunbed in prior three months/taking vitamin D supplements or photoactive medication/pregnancy/breast-feeding/smoking. The study was approved by The University of Manchester Research Ethics Committee (ref 11266), registered at www.isrctn.org (ref 99738113) and adhered to Declaration of Helsinki principles; participants gave written informed consent.

### 2.2 Skin assessments

Detailed standardised phototype assessment was performed according to modified Fitzpatrick [22]. Volunteers described their (i) propensity to burn: virtually always/sometimes/rarely/never; (ii) propensity to tan: never/light/medium/heavy; (iii) response to first occasion of 30–40 min unprotected exposure to midday June sun. Volunteers’ ethnicity, skin/hair/eye colour, and freckling presence/absence were recorded.

A spectrophotometer (CM-600D, Konica Minolta, Tokyo, Japan) recorded triplicate measurements of skin lightness (L*) from a sun-protected site (upper buttock, or upper inner arm if lighter) using the L*a*b* colour space, scale 0–100 (black-white) [23].

An individual's minimal erythema dose (MED) was assessed as the lowest UVR dose producing visually discernible erythema at 24 h, as follows. A geometric series of 10 doses (~30% increments) of erythemally-weighted UVR was applied to photoprotected skin (upper buttock, or upper inner arm if lighter) using a Philips (Amsterdam, Netherlands) TL-20W/12 lamp (280–400 nm, peak 312 nm). Thresholds in darker skin were confirmed by determining minimal flux dose, as described [4,24].

### 2.3 Simulated sunlight exposure in vivo

A single 0.8 MED of UVR was given to each volunteer using a horizontal whole-body irradiation cabinet (Philips HB598) fitted with Arimed-B (Cosmedico GmbH, Stuttgart, Germany) fluorescent tubes emitting a UVR spectrum similar to UK midday summer sunlight (95% UVA: 320–400 nm; 5% UVB: 290–320 nm). Emission was characterised and monitored as described [4]. Volunteers wore standardized T-shirt and shorts, i.e. summer clothing exposing ~35% body surface area (BSA) [4].

### 2.4 Urinary sampling and analysis

All volunteers provided a morning baseline mid-stream urine sample. Following UVR, each subsequent void was collected for five days (additional to a sample immediately pre-UVR). Samples were stored at −20 °C until analysis at Chung Shan Medical University, Taichung, Taiwan for creatinine, and 8-oxodG and 8-oxoGuo concentrations using validated LC-MS/MS methodology [18]. The limits of detection were 0.002 ng/mL for 8-oxodG and 0.003 ng/mL for 8-oxoGuo. Intraday/interday imprecisions in urine ranged from 1.4 to 5.0% for 8-oxodG and 2.9–13.7% for 8-oxoGuo; recoveries in urine were 94–101% and 109–117% respectively.

### 2.5 Statistical analysis

Outcomes were urinary 8-oxodG and 8-oxoGuo. Data were Ln-transformed for analysis. Pearson correlation coefficient examined relationship between L* and baseline 8-oxodG or 8-oxoGuo. Effect of time post-UVR and phototype on urinary 8-oxodG and 8-oxoGuo post-UVR was analysed by linear mixed-effects regression. Analyses were adjusted for repeated measurements by treating volunteers as a random effect. As 8-oxodG and 8-oxoGuo were hypothesised to increase, or increase then decrease, over the five day collection period, both time post-UVR and (time post-UVR)^2^ were explored as the independent variable.

## 3 Results

### 3.1 Volunteer characteristics

Sixty-five volunteers (mean 31 years; 34F/31 M; Table 1) participated, each providing a baseline urine sample. Fifteen of these received a UVR exposure, providing a total 460 post-UVR urine samples (Fig. 1).

**Table 1:** Characteristics of all volunteers (n = 65).

Skin type	n	Gender (F/M)	Age	L*	MED (mJ/cm^2^)	Skin colour	Hair colour	Eye colour
I	12	3/9	36 (8)	73.9 (2)	21 (5)	Light white	Sandy/red (25%)	Light blue/green/grey (42%)
							Blonde (17%)	Blue/green/grey (50%)
							Chestnut/dark-blonde (58%)	Dark blue/hazel (8%)
II	14	9/5	29 (6)	72.0 (3)	26 (4)	Light white	Blonde (7%)	Light blue/green/grey (7%)
							Chestnut/dark blonde (50%)	Blue/green/grey (57%)
							Dark brown (29%)	Dark blue/hazel (21%)
							Black (14%)	Dark brown (7%)
								Black (7%)
III^a^	19	11/8	31 (7)	69.7 (3)	42 (15)	White	Chestnut/dark blonde (22%)	Light blue/green/grey (17%)
							Dark brown (72%)	Blue/green/grey (17%)
							Black (6%)	Dark blue/hazel (33%)
								Dark brown (33%)
IV	6	4/2	30 (9)	63.1 (5)	57 (13)	Olive/light brown	Dark brown (33%)	Dark brown (67%)
							Black (67%)	Black (33%)
V	7	2/5	30 (8)	50.2 (7)	75 (19)	Mid-brown	Dark brown (29%)	Dark brown (100%)
							Black (71%)	
VI	7	5/2	31 (8)	40.5 (5)	21 (141)	Dark brown/black	Dark brown (29%)	Dark brown (100%)
							Black (71%)	

Data are mean (SD) unless otherwise stated.

a Missing information on hair and eye colour for one volunteer.

### 3.2 Baseline urinary 8-oxodG and 8-oxoGuo across phototypes

Mean baseline urinary 8-oxodG and 8-oxoGuo for each phototype are shown (Table 2).

**Table 2:** Baseline urinary 8-oxodG and 8-oxoGuo levels grouped by skin type and ethnicity (n = 64).

Skin type	n	8-oxodG (ng/mg creatinine)	8-oxoGuo (ng/mg creatinine)
I	12	3.9 (1.6)	5.6 (1.4)
II	14	3.7 (1.0)	4.9 (1.0)
III	19	4.1 (1.9)	5.5 (1.5)
IV	6	3.4 (2.3)	5.5 (2.2)
V	7	2.1 (0.6)	4.1 (0.6)
VI	6	2.0 (0.6)	3.5 (0.6)
**Ethnicity**			
White Caucasian	49	3.9 (1.7)	5.4 (1.5)
South Asian	6	1.7 (0.4)	3.9 (0.6)
Black	9	2.2 (0.6)	3.7 (0.7)

Data are mean (SD).

**Fig. 2:**
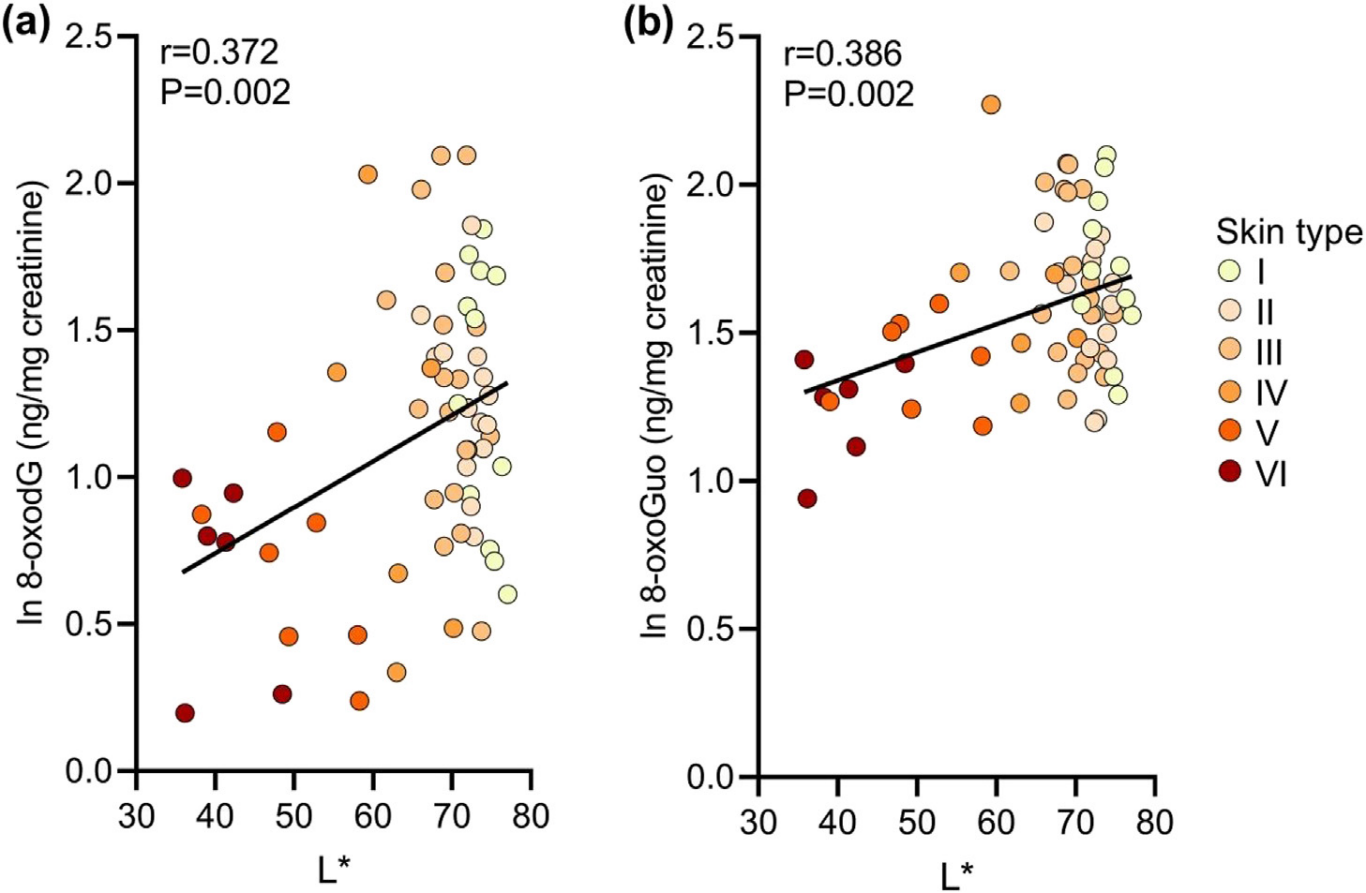
Relationship between skin lightness and urinary biomarkers of oxidative stress. Urinary levels of oxidative stress biomarkers at baseline were quantified by LC-MS/MS. A significant positive correlation was found between skin lightness L* and (a) 8-oxodG (r = 0.372, P = 0.002), and (b) 8-oxoGuo (r = 0.386, P = 0.002). Data shown for n = 64 volunteers (missing 8-oxodG data for n = 1).

Lighter skin volunteers had higher levels of both biomarkers. A linear correlation between L* and both 8-oxodG (r = 0.372, P = 0.002) and 8-oxoGuo (r = 0.386, P = 0.002) (Fig. 2) indicated that ~14% and 15% of variation in baseline 8-oxodG and 8-oxoGuo, respectively, could be explained by skin lightness.

### 3.3 Urinary 8-oxodG and 8-oxoGuo following 0.8 MED UVR exposure

A median (range) of 33 (25–36) and 32 (15–48) urine samples/volunteer were collected in the light (phototype I) and dark (phototype V/VI) groups, respectively, over the five days (Table 3). The 0.8 MED exposure equated to median UVR doses of 16.8 mJ/cm^2^ (1.68 SED; range 11–22 mJ/cm^2^, 1.1–2.2 SED) for phototype I and 72 mJ/cm^2^ (7.2 SED; range 54–130 mJ/cm^2^, 5.4–13 SED) for phototype V/VI.

**Table 3:** Characteristics of volunteers who underwent simulated sunlight exposure (n = 15).

Skin type	MED, mJ/cm^2^	UVR dose, mJ/cm^2^ (SED)	L*	Ethnicity^a^
I	12	10 (1.0)	75.35	White Caucasian
I	14	11 (1 .1)	77.07	White Caucasian
I	20	16 (1.6)	73.91	White Caucasian
I	21	17 (1.7)	72.16	White Caucasian
I	21	17 (1.7)	70.76	White Caucasian
I	28	22 (2.2)	73.61	White Caucasian
I	28	22 (2.2)	75.61	White Caucasian
I	28	22 (2.2)	72.29	White Caucasian
V	68	54 (5.4)	58.05	South Asian
V	90	72 (7.2)	52.80	South Asian
V	90	72 (7.2)	46.84	Black
V	102	82 (8.2)	49.31	South Asian
VI	83	66 (6.6)	44.98	Black
VI	163	130 (13.0)	39.02	Black
VI	205	164 (16.4)	36.18	Black

a South Asian volunteers were Indian or Pakistani; Black volunteers were Black African or Black British.

**Fig. 3:**
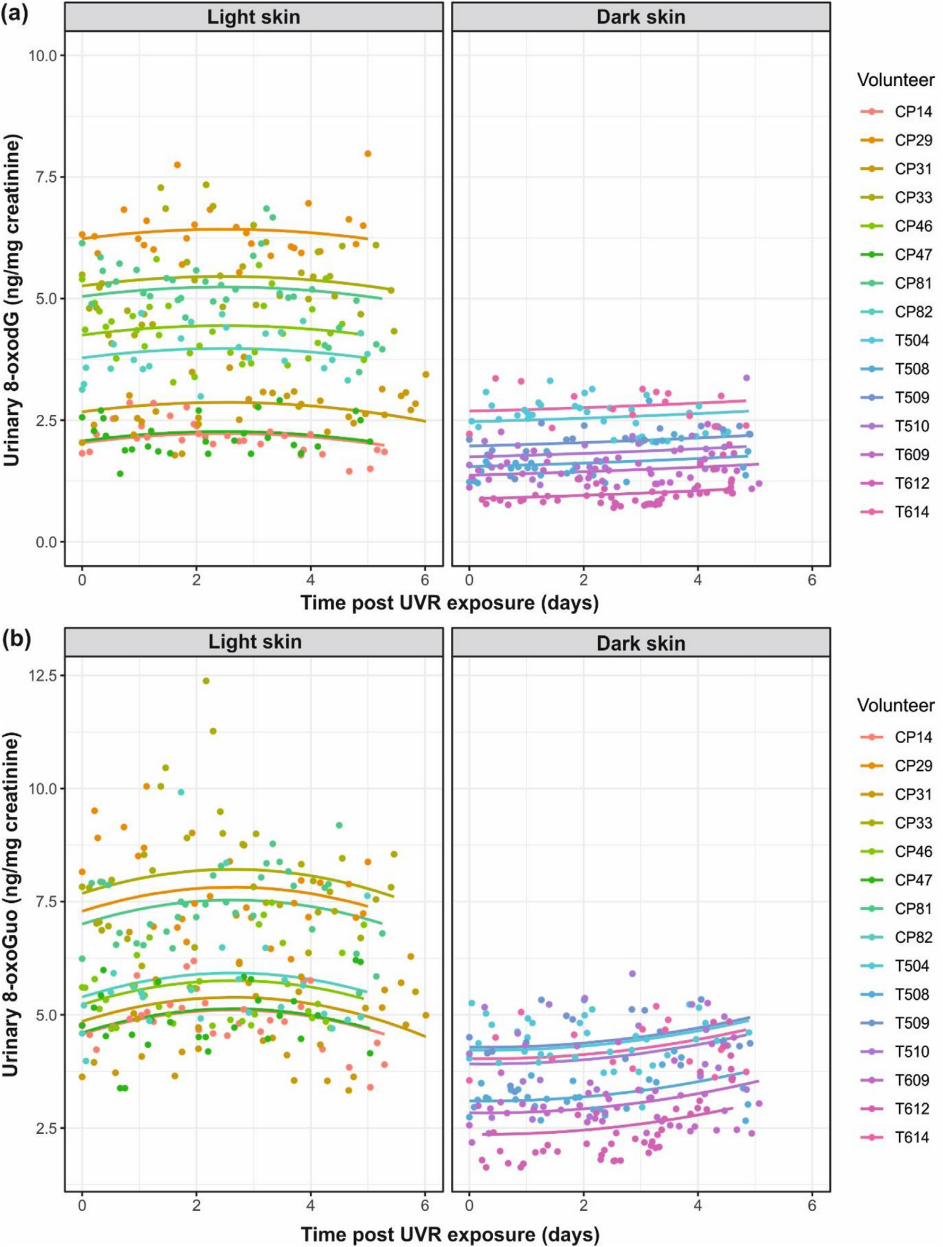
Observed points and modelled curves (one per participant) for urinary 8-oxodG and 8-oxoGuo levels following 0.8 MED of UVR. Urinary 8-oxodG and 8-oxoGuo levels were measured in every urine void for five days post-UVR. (a) A statistically significant (P = 0.01) increase and decrease in 8-oxodG occurred overall, with dark skin types at much lower values throughout. The apparent difference in curvature between skin type groups was not statistically significant (P = 0.11). (b) A statistically significant (P = 0.001) change in 8-oxoGuo levels occurred overall with lower values and a significant difference (P = 0.006) in curvature in the dark skin group. Two data-points (8-oxodG values = 24.13 and 31.38) from a skin phototype V subject were clearly erroneous and were excluded from analyses.

Baseline levels of urinary 8-oxodG (mean (SD): 4.1 (1.9) and 1.4 (0.5) respectively, P = 0.01) and 8-oxoGuo (5.7 (1.6) and 3.6 (0.8) respectively, P = 0.01) differed between light and dark groups. Post-UVR, mixed-effects regression of 8-oxodG levels overall showed initial increases, with levels returning towards baseline within the five day follow-up (P = 0.01); although this return was evident in the light group, the difference between groups was not statistically significant (P = 0.11; Fig. 3a). A very similar overall response was seen for 8-oxoGuo (P = 0.001); here, apparent difference in kinetics between groups were significant (P = 0.006), with no evidence of levels in the dark skin group decreasing during the follow-up period (Fig. 3b). No evidence of a circadian pattern in levels of oxidative species was found on autocorrelation analysis (data not shown).

## 4 Discussion

**Fig. 4:**
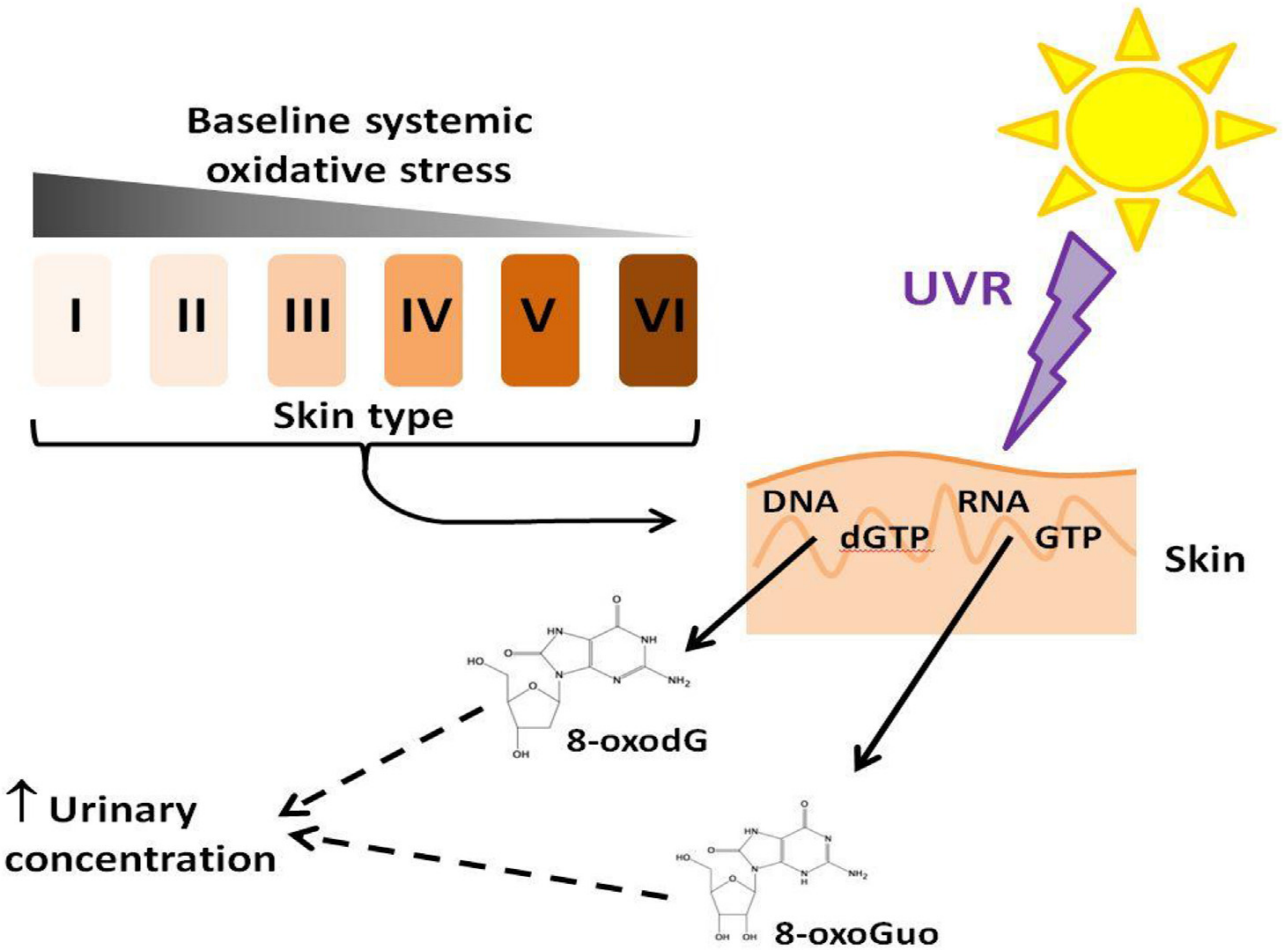
Schematic illustrating relationship of skin melanisation and UVR exposure to biomarkers of systemic oxidative stress.

Little is known of the relationship between systemic oxidative stress and constitutive skin pigmentation. Moreover, influence of cutaneous UVR exposure on oxidative stress is poorly understood. Herein, we showed, for the first time, that baseline levels of both urinary 8-oxodG and 8-oxoGuo correlate with phototype, suggesting that skin melanisation provides protection against oxidative stress. Levels of 8-oxodG were ~twice as high and 8-oxoGuo ~1.5 times higher in the lightest versus darkest skin type. To examine the impact of UVR exposure, individuals of the lightest and darkest skin received a near-sunburn dose of solar simulating UVR. Collection and analysis of every urine void for five days post-UVR revealed the single exposure to ~35% BSA was sufficient to produce detectable increases in both 8-oxodG and 8-oxoGuo in light and dark skin people (Fig. 4).

In a pilot study, higher urinary 8-oxodG in individuals of phototype II than V was incidentally observed [25]. Our investigation in groups of individuals over the entire phototype range has now revealed a significant positive correlation between skin lightness (L*) and baseline 8-oxodG (r = 0.372, P = 0.002) and 8-oxoGuo (r = 0.386, P = 0.002). Thus, melanisation level explains ~14–15% of the variation in baseline systemic oxidative stress level, as indicated by 8-oxodG and 8-oxoGuo. This intriguing finding may reflect profound underlying patho-physiological differences between people of different phototypes.

Genotype and cultural differences may also contribute. It was pivotal to this discovery that individuals were recruited from the same location, and assessed under the same conditions, as environmental stressors differ across locations/seasons, with inter-laboratory variation in measurement of these biomarkers being considerable [26].

Skin melanin content shows strong negative correlation with non-invasively measured skin lightness [4]. Melanin scavenges ROS, and decreases oxidatively-generated damage to DNA, proteins and lipids *in vitro* [27], this protective function mostly attributed to blue-black eumelanin content [28]. Analogous to our findings, the size of the eumelanin-containing facial mask of the bird *Geothlypis trichas* correlates with resistance to oxidative stress in an *in vitro* assay [29]. Red-yellow pheomelanin is also found in human skin, generally with lower proportion of pheomelanin:eumelanin with greater pigmentation [2,30]. Pheomelanin generates ROS, decreases antioxidant levels and is prone to photosensitization [[31], [32], [33], [34]]; lower levels of biomarkers of systemic oxidative stress in darker skin people might reflect lower pheomelanin as well as higher eumelanin levels.

Since UVR can cause oxidatively-generated damage through ROS generation, we performed a post-UVR time-course study. This revealed that a solar-simulating UVR exposure provoked an increase in urinary 8-oxodG and 8-oxoGuo across light and dark skin types (P = 0.01, P = 0.001 respectively). In light skin individuals, levels of both species showed initial increase followed by return to baseline, with peak ~ day three. This pattern of response was less evident for 8-oxodG in dark skin individuals, while for 8-oxoGuo the kinetics significantly differed from light skin individuals, with no evidence of decrease during the five days. This indicates a longer period of cutaneous nucleic acid damage repair in the darker skin group, potentially reflecting the higher absolute UVR dose given and/or intrinsic difference in repair kinetics.

Studies of urinary biomarkers of oxidative stress following UVR exposure are scarce. Urinary 8-oxodG was examined following single-dose whole body photochemotherapy (psoralen-UVA; PUVA); urinary 8-oxodG peaked ~ day four [35]. However, PUVA's phototoxic reaction differs from sunburn. Pilot work exploring impact of low level UVR exposures showed no impact on urinary 8-oxodG [25]. However, the current, personalised UVR dose, close to the sunburn threshold (0.8 MED, median SED 1.68 and 7.2 in phototypes I and V/VI respectively) to ~35% BSA provided a level of insult that induced oxidative stress. Sun exposure recommendations are to keep below personal sunburn threshold; accordingly we UVR-exposed volunteers according to individual threshold. Pivotally, we mimicked natural conditions (UVR emission close to ambient summer sunlight: 5% UVB, 95% UVA; volunteers wearing summer clothing). In contrast, personal exposure to ambient UVR is extremely low in winter-time at 53.5°N (~0.1 SED/week to ~8% BSA) [36].

In conclusion, this original work notably reveals a linear relationship between skin lightness and baseline 8-oxodG and 8-oxoGuo levels, which we propose is principally due to an antioxidant effect of melanin. Further, sub-sunburn cutaneous UVR exposure can cause detectable levels of oxidatively-generated damage to nucleic acids, so simple avoidance of visible skin redness is insufficient to avoid tissue damage, and solar UVR may contribute to systemic oxidative stress during summer-time. Biomarkers 8-oxodG and 8-oxoGuo behaved similarly in response to UVR, suggesting similarity in origin e.g. the nucleotide precursor pools, while differences in their kinetics were apparent between light and dark skin types.

Future studies could examine quantity/location of oxidatively-generated damage in the tissues of light and dark skin people, together with DNA repair capacity. Further perspectives for research include a comparison of the responses of oxidative stress biomarkers derived from other groups of compounds, e.g. lipid/protein that occur in urine. The finding of lower systemic oxidative stress levels with greater melanisation has important implications for human physiology and health.

## Declaration of competing interest

The authors declare no conflict of interest.
